# Analysis of Free Online Physician Advice Services

**DOI:** 10.1371/journal.pone.0059963

**Published:** 2013-03-26

**Authors:** Raphael Cohen, Michael Elhadad, Ohad Birk

**Affiliations:** 1 Department of Computer Science, Ben-Gurion University, Beer-Sheva, Israel; 2 Genetics Institute Soroka Medical Center and The Morris Kahn Laboratory of Human Genetics, National Institute for Biotechnology in the Negev (NIBN), Ben-Gurion University, Beer-Sheva, Israel; University Hospitals of Geneva, Switzerland

## Abstract

**Background:**

Online Consumer Health websites are a major source of information for patients worldwide. We focus on another modality, online physician advice. We aim to evaluate and compare the freely available online expert physicians’ advice in different countries, its scope and the type of content provided.

**Setting:**

Using automated methods for information retrieval and analysis, we compared consumer health portals from the US, Canada, the UK and Israel (WebMD,NetDoctor,AskTheDoctor and BeOK). The evaluated content was generated between 2002 and 2011.

**Results:**

We analyzed the different sites, looking at the distribution of questions in the various health topics, answer lengths and content type. Answers could be categorized into longer broad-educational answers versus shorter patient-specific ones, with different physicians having personal preferences as to answer type. The Israeli website BeOK, providing 10 times the number of answers than in the other three health portals, supplied answers that are shorter on average than in the other websites. Response times in these sites may be rapid with 32% of the WebMD answers and 64% of the BeOK answers provided in less than 24 hours. The voluntary contribution model used by BeOK and WebMD enables generation of large numbers of physician expert answers at low cost, providing 50,000 and 3,500 answers per year, respectively.

**Conclusions:**

Unlike health information in online databases or advice and support in patient-forums, online physician advice provides qualified specialists’ responses directly relevant to the questions asked. Our analysis showed that high numbers of expert answers could be generated in a timely fashion using a voluntary model. The length of answers varied significantly between the internet sites. Longer answers were associated with educational content while short answers were associated with patient-specific content. Standard site-specific guidelines for expert answers will allow for more desirable content (educational content) or better throughput (patient-specific content).

## Introduction

Online health portals are a popular source for medical information: in two different 2003 surveys, 40–65% of internet users in the US reported searching for health information online at least once a year and 9–30% monthly [1], [2]. A subsequent survey in 2005 [3] showed that 79% of internet users have sought health information online. Hesse *et al.* [4] used data from the Health Information National Trends Survey to show that a large percentage of the online population (internet users) go online for health advice before turning to their physicians. In Canada, the use of internet for medical searches was reported at 64% during 2010 [5]. Similarly high internet eHealth literacy was also reported for Israel [6].

Health related information on the internet is available in the form of articles and medical encyclopedias addressing specific topics [7]. The quality, accuracy and reliability of such online eHealth resources are controversial. Individuals seeking answers to specific questions may be liable to find information that is either misleading or irrelevant to their specific case. It is difficult for laypersons to sift intelligently through the vast amounts of available information to find answers truly relevant to their case.

In contrast to general purpose encyclopedias, patient forums have emerged as a popular source of relevant information. Such forums provide information and support by patients with a specific condition (*e.g.,* breast cancer forum [8], diabetes forum) with some including over a million registered users [8]. Online support forums are more accessible to the lay user as information and advice is explained by other laymen suffering from the same disease. The quality of advice in support forums may vary: while breast cancer forums were found to be extremely accurate [9], other studies show this information source suffers from variable information accuracy even in moderated forums [10].

More generally, Choi *et al*. [11] proposed a typology of online Question-Answering (QA) forums into four categories: (1) community-based forums (such as the patient forums described above), (2) collaborative QA (such as WikiAnswer, which produces sites quite similar to an encyclopedia), (3) expert-based QA and (4) social QA (using Twitter or Facebook for asking a question). Another type of QA is automatic answering systems (see [12]). However, these automatic systems mostly use MEDLINE which is not suited for the lay user and cannot process extremely complex questions in natural language. Expert QA, is highly relevant to the eHealth field. We have identified several resources providing QA services by expert physicians. Consumer Health websites such as WebMD, NetDoctor, BeOK and AskTheDoctor, allow a user to post a question and obtain an answer by a physician certified by the website. In contrast to non-interactive information pages such as an encyclopedia, expert QA sites allow patients to obtain relevant and accurate information. Answers offered in such sites are far more relevant for the lay-person than sifting through the professional information posted in a disease description page on an encyclopedia site or through automatic symptoms-based search engines. In contrast to patient forums, these expert QA services provide information that is reviewed by formally trained and licensed specialized medical practitioners. This information is likely to be more accurate than an answer in a support group in an online forum, where the level of expertise of the answering party is unknown (chronic or serious conditions such as cancer may be the exception as patients are forced to self-educate about their disease). In a study of the accuracy of information learned from expert networks for occupational safety and health, use of expert networks provided better quality of answers than encyclopedia lookup [13]. Kummervold and Johnsen [14] showed that in email communication with their patients, physicians were able to answer 95% of the questions in 2.2–2.7 minutes, regardless of question length.

Whereas quality and trust levels of both encyclopedia and patient forum consumer health sites have been studied in the past, interactive online physicians’ answering services have not been analyzed. In this study we compare the length, content (percent of medical terms within the response), response times, amount of physicians’ answers as well as the frequency of questions in the various categories in three dominant consumer health websites in English from the US (WebMD), UK (NetDoctor) and Canada (AskTheDoctor) and one in Hebrew from Israel (BeOK). Furthermore, we assess delineation of two types of online physician advice approaches: services providing broad answers which may be useful for educating many patients, versus services providing shorter focused answers likely to be useful only to the patient asking the question.

## Methods

### Online Clinical Q&A Services

We collected all the web pages containing physicians answers from four websites supplying QA services: WebMD, online US health giant with net worth of 2.5 billion dollars; AskTheDoctor, Canadian-based question answering website; NetDoctor, a European-based online health information website (focusing on its UK subsidiary netdoctor.co.uk); and BeOK.co.il, an Israeli-based medical information website. WebMD, NetDoctor and BeOK are consumer health portals ranked as highly popular in their locale according to Alexa.com. AskTheDoctor specializes only in QA and has less traffic than the overall consumer health portals.

Compensation model: WebMD and BeOK both use volunteer physicians with the exception of paid “guest experts” who contribute only for a brief period of time. Experts’ names are displayed in the forum information even when not browsing their answers. Contribution to the site, therefore, provides publicity and builds reputation for the experts. In AskTheDoctor and NetDoctor, the compensation model is not disclosed, and the physicians’ names are not displayed.

### Data Extraction

From each of these websites, all web pages containing physician-certified answers were collected. The web pages were extracted using automatic scripts implemented in Python (scripts are available at www.cs.bgu.ac.il/~cohenrap/consumer-health-scripts.zip). Data were extracted during July 2011. In WebMD and BeOK, where a single question can be followed up by a threaded discussion containing several answers, only one answer was collected for each question.

We extracted and computed the following information for each site: number of question/answer pairs per year, length of answers, density of medical terms in the answers, delay between question and answer, topics of the question-answer pairs and distribution of answers per contributing physicians. Finally, we compared the compensation model offered to contributing physicians.

Metadata was extracted for each answer: date of physician answer was extracted from the answer webpage for comparison by year and topic of the forum. In BeOK and WebMD the questions/answers are in a forum structure allowing the extraction of physician names and date of the question (for calculating response time). As access to information in NetDoctor and AskTheDoctor was limited, our analysis of those two sites was limited only to data regarding topic distribution of questions and answer rate in Canada and the UK.

### Data Coding, Verification and Analysis

Topics of the question-answer pairs were coded based on the subgrouping within the websites. Where different terminology was used at the various sites for overlapping categories (for instance, “obstetrics” vs. “pregnancy”), such overlapping categories were unified. Sub-categories were also unified: for example, “epilepsy” and “Parkinson” were both labeled as “neurology”. This coding was performed by one of the authors, a medical doctor.

The amount of medical content of the messages was compared by mapping the notes to UMLS (Unified Medical Language System), a medical vocabulary [15] by the NLM. For the English websites (AskTheDoctor, NetDoctor and WebMD) we used HealthTermFinder [16], an automatic medical term annotation program that identifies UMLS [15] terms in free text. The content of the notes was pre-processed to identify shallow syntactic structure: part-of-speech tagging with the GENIA tagger [17] and phrase chunking with the OpenNLP toolkit [18]. HealthTermFinder recognizes named-entities mentioned and maps them to semantic concepts in the UMLS. It was tested on a gold standard of 35 clinical notes from the Columbia University Medical Center. The notes contained 2,589 mentions of clinical entities, corresponding to 1,056 unique entities, as recognized through gold-standard manual annotation. When compared with state-of-the-art MetaMap [19], HealthTermFinder identified the mentions with significantly better success: 88.55 (.013 95% CI) F-measure vs. 77.54 (.0165 95% CI) for MetaMap for exact matches of mentions [16]. In a separate study, HealthTermFinder has been shown to have sensitivity of 91% and specificity of 86% [20] for identifying medical concepts in Pubmed abstracts.

BeOK answers were analyzed using an in-house Hebrew NLP pipeline for mapping to UMLS [21]. Manual evaluation of this pipeline on 100 sentences from BeOK (containing 205 medical terms) answers, found sensitivity of 84% and specificity of 96.9% (the number of medical terms identified automatically was slightly lower than the number found manually).

Due to the agglutinative nature of Hebrew (prepositions, definite articles and possessive pronouns are agglutinated to the word) it is not possible to accurately compare response lengths as a single word in Hebrew may be translated into 2 or 3 words in English. This linguistic aspect of Hebrew results in text which is typically ∼30% shorter in Hebrew compared to the corresponding English translation.

The extracted concepts were used for quantifying the density of expert medical terms used in a document. To compare between the different websites, we normalized the number of concepts with the number of words in the message (*i.e.,* we report the density of medical terms as the ratio of answer length/number of medical concepts).

The lengths of physician answers per each physician were analyzed for WebMD and BeOK. Only physicians who contributed more than 100 answers were assayed. Correlation between question and answer lengths was calculated using Pearson’s product-monument correlation test. Response time was calculated by using the timestamp of the first answer by an expert to the patient’s question.

To test the association between answer length and answer type (Educational/Patient Specific), we extracted short and long answers from WebMD and BeOK. For WebMD, we defined short answers as those containing 30–50 words while answers harboring 120–170 words were defined as long ones. For BeOK, we defined short answers as those containing 5–45 tokens and long answers as those with 110–170 tokens (before word segmentation). The slightly shorter length in Hebrew reflects the agglutinative nature of the language. Two annotators, fluent in both Hebrew and English, tagged each text as either:

- ***Educational***: answer aimed to teach and provide information about the conditions described in the question in a general manner
- ***Patient Specific***: provides a specific solution/advice to a specific patient, not likely to be useful to others
- ***Not sure***

Answers which at least one of the annotators categorized as “*not sure”* were removed from the analysis. Inter-Annotator Agreement and Cohen’s Kappa [22] were calculated. Proportions of the answer type in each length category were calculated using only the answers for which both annotators were in agreement.

To address the question of timing of the use of QA services, before or after visiting a physician. 100 questions from 20 forums in WebMD were annotated as: (i) asked before visiting a physician (ii) after visiting a physician and (iii) not clear.

## Results

### Websites Description

We identified 3 formats for websites of free expert medical advice.

1. **Closed Q/A** – Users submit a question and the question/answer pair is posted online. This is the format of NetDoctor and AskTheDoctor.
2. **Expert Forum** – In this format, users post questions in an online forum. The physicians answer all the forum threads. This format is used by BeOk.
3. **Expert Moderated Community Forum** – This format, used in WebMD, allows physicians to comment on questions posted in community forums. Most of the answers are provided by non-expert community members and the physician may choose an interesting or complicated question to address or intervene in an active discussion.

In both forum formats (formats 2 and 3 above), physicians and patients may interact, further clarifying the question or answer or asking for more information.

The following examples illustrate the type of QA pairs found in the sites:

Short Q/A from WebMD:

**Q** (WebMD Aug 15): “*went in for my twenty week ultrasound and they said the measurements put my due date 9 days longer…They also said baby was weight about 9 oz is this ok?*”.

**A** (Aug 19, 28 words): “*The 9 days is within the error of the ultrasound equipment. It is not different. The weight of the fetus is within normal limits as well. Good luck*”.

Long answer from WebMD:

Q (WebMD Jul 11): “*My husband is having a very severe migraine and from what I can find it sounds like a hemiplegic migraine. He had all the stroke symptoms but everything checked out ok. It’s been 6 days and he is still having the bad headache, nausia and a little light sensativity. Is it normal for a migraine to last 6 or more days? Is it normal for the hemiplegic migraine to last that long?*”.

A (Jul 16, 166 words): “*Migraines can last for hours, days or weeks. Hemiplegic migraines are no different, and the weakness on one side of the body can last as long as the headache. The longest I’ve personally seen is a patient who had weakness from hemiplegic migraine that lasted for 4–6 weeks each time it happened (fortunately this patient had the hemiplegic migraines only 1 time a year). Hemiplegic migraines are not reported to cause damage to the brain Headache weakness on one side of the body damage on MRI scan is called a STROKE, not hemiplegic migraine.*

*Hemiplegic migraine is diagnosed by at least 2 MRI scans of the brain during the migraine (with weakness) that show no brain abnormalities and no stroke.*

*Some types of hemiplegic migraine are strongly inherited, and there is actually a genetic test for that type of hemiplegic migraine. The test doesn’t help use treat the problem though.*

*Most of the time, the regular migraine prevention medications are effective for preventing hemiplegic migraines also.*”

Short Answer from BeOK (translated from Hebrew).

**Q** )BeOK Sep 11): “*Dear Drs, after second trimester screening, week 21+4, what does this result mean:???? HC/AC: 1.26, slightly above the range of normal 1.24. Thank you*”.

**A** (Sep 12, translated, 21 words): “*Good evening, according to this report it is normal, it says that head to abdomen ratio is slightly large. Totally meaningless.*”

### Topics of Available Answers

**Figure 1:**
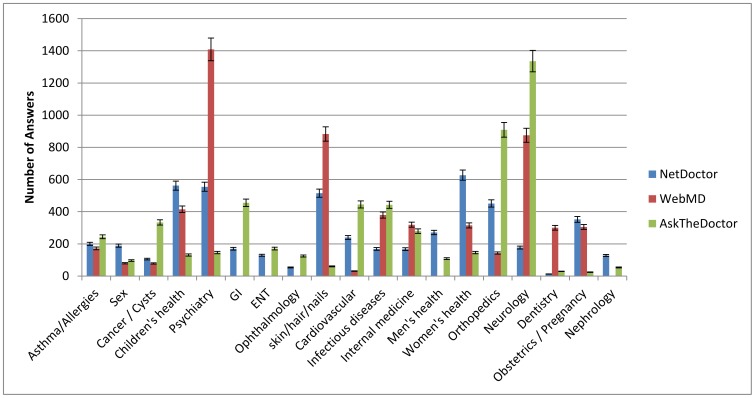
Number of questions/answers by topic.

**Figure 2:**
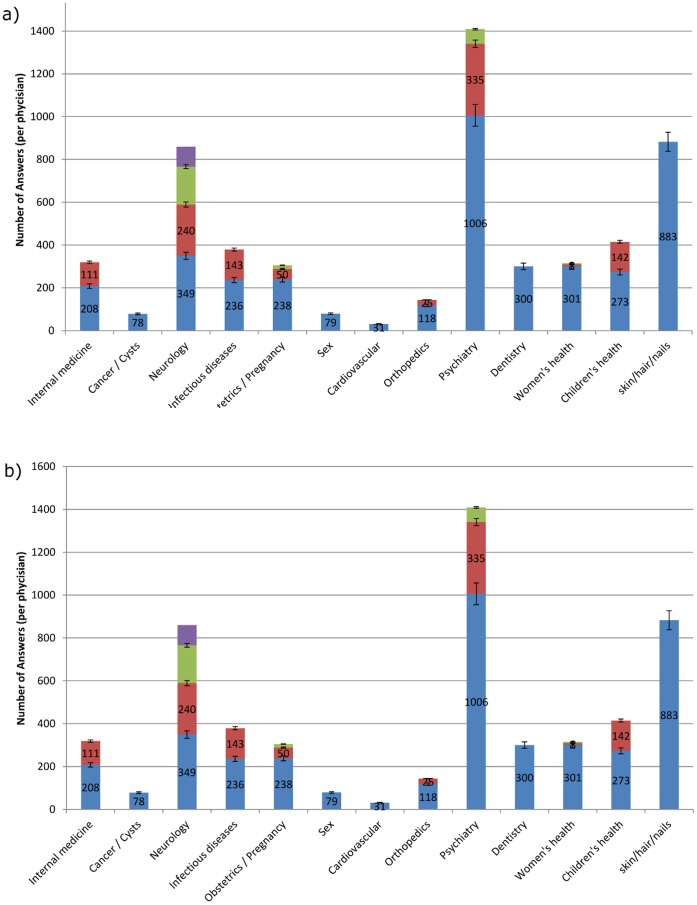
Topics by doctor contribution in BeOK (a) and WebMD (b). For each topic the physicians contributing to that topic are marked in different colors.

In order to compare topic coverage of physicians’ answers in the various websites, topics dealt with in each website were classified into 19 categories (see Figure 1), manually crafted to best represent the data. The amount of information varied between the different categories in the different sites. As shown in Figure 1, in WebMD the majority of questions and answers were related to the Psychiatry and Skin/Hair/Nails topics, while most answers in AskTheDoctor focused on Neurology and Orthopedics and in NetDoctor on Women’s Health-related questions. In BeOK (Figure 2a) the most popular topics were Obstetrics/Pregnancy (24,526 answers) and Cancer (15,158 answers). While the coverage of most topics in BeOK is greater than in the other 3 websites with an average of 5,759 answers per topic, some topics such as Neurology, Nephrology and ENT were under-represented. Overall, questions regarding psychiatry, neurology and skin/nails/hair were the most common in all websites studied.

### Physician Contribution

On average, each individual doctor in WebMD contributed overall 212 answers over the entire period sampled on the sites (Figure 2b). The number of answers provided by different doctors varied greatly between individuals with a standard deviation of 241 answers (ranging from 6 to 1006 answers). The three top contributors (1006, 883 and 335 answers) account for a third of the total amount of available answers (total of 2,224 answers given by 3 physicians). A similar trend could be seen in BeOK (Figure 2a), with 1–5 excellent contributors in each topic (6 different physicians answered 1,000 or more questions regarding cancer) but with many smaller contributions as well (18 contributors in Cancer). Comparative analysis of a single year (2010) showed that in BeOk, 101 physicians answered 292 questions each on average, yet the top 3 contributors answered 6,453 questions, 21% of all questions answered. Similarly, in WebMD each physician answered 127 questions on average, yet the top 3 contributors answered 1,763 questions, which were 51% of questions answered in 2010.

### Information Content

We assessed the amount of professional medical terms used in the text. In BeOK, 29% of the words were identified as medical concepts (CI 28.4%–29.9%), compared to 25.7% (CI 95% 25.5%–25.9%) in AskTheDoctor and ∼21.5% in WebMD and NetDoctor (see Table 1).

**Table 1:** Characteristics of physician response in the different websites: answer length and proportion professional medical terms in the answer.

Website	Average response length (number of words) [CI 95%]	Proportion of words using medical terminology [CI 95%]
NetDoctor (UK)	190 [187–193]	21.4% [21.3%–21.4%]
AskTheDoctor (Canada)	121 [119–123]	25.7% [25.5%–25.9%]
WebMD (USA)	109 [107–111]	21.8% [21.7%–22.0%]
BeOK (Israel)	35 [34.6–35.7]	29.9% [29.7%–30.0%]

### Answer Length and Type: Educational vs. Patient-specific

Answer length varied between the websites with NetDoctor being the longest with 190 words on average (CI 95% 187–193) and BeOK being the shortest with an average of 35 words per answer (CI 95% 34.6–35.7).

Two annotators categorized the answers as either educational or patient-specific. The annotators had agreement of 83% (kappa 0.65) for BeOK answers and 79% (kappa 0.45) for WebMD answers. Short answers were likely to be *Patient-Specific* while long answers were likely to be *Educational* (Table 2). Since BeOK contains mostly short answers (median length of 15 words), we surmise that it leans towards *Patient-Specific* answers. In WebMD, we see a more balanced division with a median length of 90 words, suggesting similar prevalence of both types of answers.

**Table 2:** Characteristics of physician response in the different websites: answer length and proportion professional medical terms in the answer.

Website	Short Answers [95% CI]	Long Answers [95% CI]
WebMD	82% *Patient Specific* [62%–93%]	70% *Educational* [57%–88%]
BeOK	80.8% *Patient Specific* [67%–89%]	73% *Educational* [55%–86%]

### Response Type by Physician

**Figure 3:**
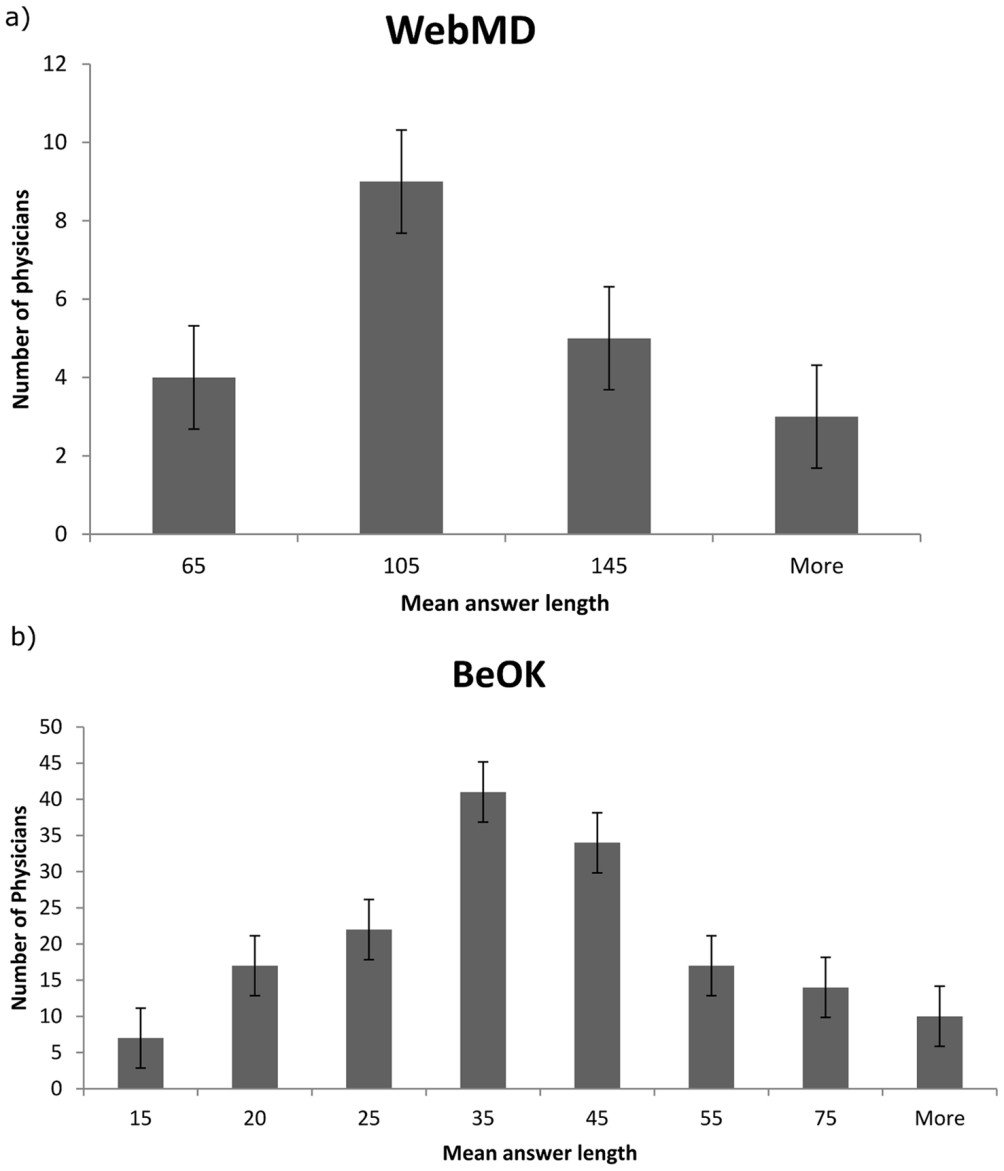
Histograms of mean answer length by physician. (a) WebMD physicians (b) BeOK physicians.

To better understand the length differences between sites, we analyzed answer length by physician. In WebMD, 21 physicians were found with more than 100 answers; in BeOK, 162 physicians passed the cutoff. We observe high variance in the mean answer length between physicians. In WebMD, the standard deviation is 31 (mean of 103 words with minimum of 60 words and maximum of 160 words). In BeOK, the differences are greater with standard deviation of 20 (mean of 38 words with minimum of 10 words and maximum of 132). This variance is also present within physicians answering questions on the same topic (in gynecology, we found 3 physicians with mean answer lengths of 10, 18 and 24; in the ENT topic, we found one physician with mean answer length of 18.5 words and another with 62). See Figure 3 for histograms of the two distributions.

These observations indicate that each physician has a preferred style of answer – educational or patient-specific, regardless of the topic of the question.

### Correlation between Question Length and Response Length

We tested for Pearson correlation between questions and answers. Weak correlation was found for all websites (0.17–0.29). See Table 3. This observation indicates that while physicians have a preferred style they also take the complexity of the question into account.

**Table 3:** Correlation between question length and answer length.

Website	Pearson product moment correlation coefficient	95% Confidence Interval
AskTheDoctor	0.17	0.151–0.199
BeOK	0.19	0.187–0.193
WebMD	0.25	0.229–0.272
NetDoctor	0.29	0.273–0.321

### Response Time

In WebMD, the mean response time is 139 hours (CI 95% 117–162 hours), 36% of the answers came within the first 24 hours. In BeOK, the mean response time was 43 hours (CI 95% 42–45 hours), 62% of the answers arrived in less than 24 hours. No correlation has been found between answer lengths and response time (Pearson’s correlation of 0.001 in BeOk and 0.03 in WebMD).

### Amount of Information Available

**Figure 4:**
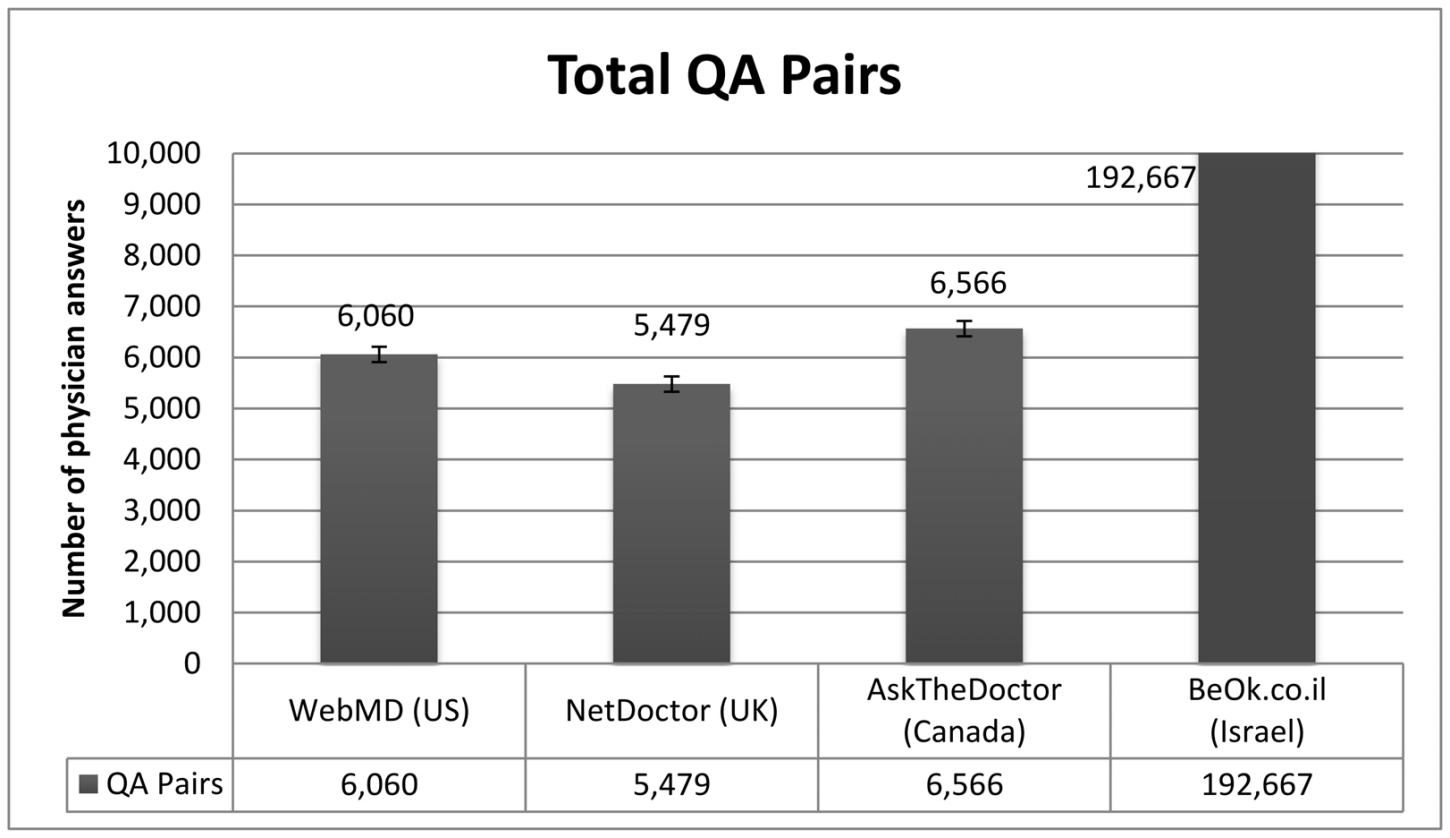
Number of question/answer pairs in each website (note that BeOK data are beyond the scale of the figure).

**Figure 5:**
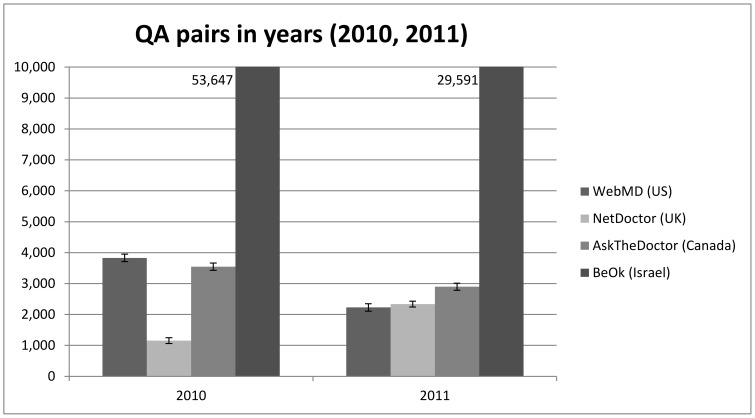
Number of question/answer pairs in each website by year (note that BeOK data are beyond the scale of the figure).

To date, 6,060 questions were answered in WebMD, 5,479 in NetDoctor, 6,566 in AskTheDoctor and 192,389 in BeOK (see Figure 4). In the first half of 2011 (Figure 5), 2,229 questions were answered in WebMD, 2,336 in NetDoctor 2,898 in AskTheDoctor and 29,591 in BeOK.

### Timing of Questions: Before or after Physician Visit

Of the 100 questions assessed, 48 (38%–57% CI 95%) were asked prior to a physician visit, 45 following it and 7 unclear. A common reason stated for questions asked prior to physician visit was length of wait for the doctor appointment and lack of insurance.

## Discussion

### Principal Findings

We found a significant difference between the number of answers in the Israeli BeOK service as compared to the three English services including the much larger WebMD consumer health portal (BeOK published 10 times more answers than WebMD over a similar time period).

We found that the quantitative criterion of answer length provides reliable characterization of the answers as either broad educational vs. patient-focused tailored answers. Major differences were observed in the type of content provided in the sites: WebMD’s expert content was characterized by longer educational answers to select questions while most questions were left to the forum community. In contrast, BeOK included many more patient-focused answers than the English sites. BeOK’s experts answered all of the questions asked, yet with shorter specific answers which were less likely to educate other patients. This difference in content type (*Patient-Specific vs. Educational)* also affected the response time, with 64% of the answers in BeOK appearing within the first 24 hours, compared to 36% of the expert answers in WebMD. It is noteworthy that many educational answers seemed to contain cut-paste sections from other information resources as well as references, suggesting more effort was made in gathering the information. It was apparent from the sites that the decision to use either type of answer corresponds to the personal style of each contributing physician.

### Study Limitations

The methods used in this study are retrospective, examining available online data. The study covers the period from 2002 to July 2011 on publically available Web sites. We have not had any direct contact with the commercial providers of these sites. NetDoctor and AskTheDoctor do not supply most of the information available in WebMD and BeOK such as physicians’ names, expertise status, answer time or the ratio of questions answered. As of September 2012, AskTheDoctor reported an overload of questions (800–1,000) a day and directed the patients to an online paid answering service for quick answers. In NetDoctor, the question answering section is not an important feature of the website, and it is complicated to find for users. We, therefore, concentrated most of our analysis on WebMD and BeOK and used the other two sites in order to analyze topic distribution of questions and answer rates in Canada and the UK. Further study of the impact of online physicians’ advice would require a wide survey of patient exposure to online advice and its impact.

We did not include qualitative and relevance assessment of the provided expert answers. Such assessment would require recruiting expert physicians for evaluating the answers in different forums, which is beyond the scope of this study. Moreover, it would be complex to determine that experts reviewing the professional quality of other experts are better than the experts giving the advice and are suited to criticize their professional quality.

### Implications of Findings

The availability of online expert-QA medical advice by specialized physicians is of utmost importance, especially in light of the torrents of often irrelevant, inaccurate and imprecise medical information streaming through the many medicine-related websites and forums. Even the accurate and reliable information offered by online encyclopedias and well accredited websites is often misused and misinterpreted by laymen, lacking the basic knowledge in the relevant field to sift wisely through the information in search for an answer truly relevant to their specific case. Community forums such as the breast cancer forum can help resolve much of that noise, but educated patients are more likely to be found for such chronic sever/chronic condition where patient are often forced to self-educate.

Our observations indicate that expert physician answers can be made available in a variety of fashions. In each of the 4 countries we investigated, major private companies can provide free expert QA services to the public at large. The sites accumulate a large collection of expert QA over the years. The voluntary contribution model used in WebMD and BeOK seems to provide sufficient incentive to gather an active community of expert physicians. One reason for providing expert answers in these forums is gaining online publicity for the physician’s private practice.

The typology of answers is important (*Patient Specific* versus *Educational)*: we expect search engines and data mining tools should apply different techniques to deal with patient-specific and broad educational answers. We also believe that the service providers should draft guidelines for the contributing physicians to recommend either type of content for their sites, according to medically-informed criteria.

We found that the most popular topics varied between the sites, with the majority of questions overall being in the fields of psychiatry and neurology, followed by skin/hair/nail (relating to esthetics) and obstetrics and gynecology/women’s health and cancer. Questions in pediatrics, sex, GI, ENT, ophthalmology, cardiovascular, infectious diseases, men’s health, dentistry and nephrology were far less common. Interestingly, in few categories, there were major differences between the different countries: for instance, questions relating to skin/hair/nails were common in the UK (NetDoctor) and US (WebMD) sites, while in the Canadian and Israeli sites questions in this category were rare. Questions relating to sex were common in the Israeli site but not in the other sites. Such differences might be at least in part due to cultural differences between the countries. One of the major advantages of online medicine in comparison to standard physician care is the anonymity of the patient. This is well reflected in the fact that questions in psychiatry are one of the most common topics in all four sites, and that questions regarding sex are frequent in BeOK.

While several physicians were available in each category in most websites, it was 1–2 physicians in each category that carried most of the burden in each category in each site. The effect seems to be self-feeding: it is plausible that having an active physician in a category leads to a further and wider influx of questions.

### Future Work

There are yet many further questions to be investigated in this arena. For instance, it is yet unclear how would these online services impact on patient-clinician relationships; whether doctors would welcome these services; how would these services sustain in the long term; Whether patients are more likely or less likely going to visit a doctor after using this service; how does having a healthcare professional’s opinion affect the patients’ relationship with their regular doctors? how can and should society govern or regulate the safety and quality of the advice provided, and accordingly, what are the legal implications if wrong advice is given, or if advice is misinterpreted? Those and many other questions are beyond the scope of this study and are yet to be addressed.

Israel stands out in that the number of answers annually is more than 10 fold higher than in the main portals serving much larger countries. This might be due to a significantly higher number of contributing physicians in the Israeli portal, as well as the fact that Israel is one of the top countries worldwide in computer literacy, internet usage, and incorporation of novel technologies. The small geographic distances in Israel makes this method of gaining publicity for private consultation more effective as most of the patients are likely to be able to reach and consult with the advising physician. A more in-depth analysis of this surprising difference in number of published answers should be pursued in the future.

### Conclusions

The length of answers varied between the internet sites. In this regard, the Israeli BeOK site is unique in that the answers are nearly 3 fold shorter than in the other sites, which is also likely conducive to the ability of physicians in this site to be more prolific and more responsive (shorter response time). We hypothesize that shorter answers indicate a preference for patient-specific answers vs. broad educational answers in this site.

In summary, we have shown that interactive sites of expert QA physician advice are an emerging arena alleviating some of the pitfalls in the field of online consumer health. Shorter, concise answers by a small number of dedicated physicians, focusing on fields of major interest, enable high throughput. The format varies between various internet sites: in WebMD, and partly in BeOK, unlike NetDoctor and AskTheDoctor, physician experts operate as moderators in patient forums. The combination of community advice with expert moderation produces high-quality timely content for patients, while requiring less effort from the experts.

We distinguished two models of online physician advice – providing either broad educational or patient-focused answers. Both models may be adopted by HMOs or public health institutions to disseminate reliable medical information. Guidelines for the expert physicians should be formed defining the desired type of answer as well as quality measures for each type.

Questions of legal liability regarding each of these models have not yet been seriously challenged and will need to be resolved in due course. Further issues of efficient search engines for these types of data and taking advantage of the different nature of the information for research purposes still remain to be addressed.
